# Case Report: A rare pediatric case of B-cell lymphoblastic lymphoma presenting as an isolated renal mass with *EWSR1::FLI1* translocation and germline *CHEK2* variant

**DOI:** 10.3389/fped.2025.1569506

**Published:** 2025-06-24

**Authors:** Katelyn P. Daniels, Kim E. Nichols, Arti S. Pandey, Gabriela Gheorghe, Sara Helmig, Kevin Garrett, Asim K. Bag, Hiroto Inaba, Raul Ribeiro

**Affiliations:** ^1^ Department of Oncology, St. Jude Children’s Research Hospital, Memphis, TN, United States; ^2^ Department of Pathology, St. Jude Children’s Research Hospital, Memphis, TN, United States; ^3^ Department of Radiology, St. Jude Children’s Research Hospital, Memphis, TN, United States

**Keywords:** B lymphoblastic lymphoma, renal mass, *EWSR1::FLI1* translocation, *CHEK2*, Wilms tumor

## Abstract

We describe a 5-year-old boy who initially presented with a large left renal mass, suspected to be Wilms tumor (WT). However, biopsy results revealed B-cell lymphoblastic lymphoma (B-LBL) manifesting as an isolated renal mass. Tumor transcriptome analysis identified an *EWSR1*::*FLI1* fusion, with breakpoints distinct from those typically associated with Ewing's sarcoma. Other somatic pathogenic variants affecting *WT1*, *ETV6, SETD2, ADD2, EZH2, PRDM2,* and *NF2* were identified*.* The patient also carried a germline *CHEK2* variant of unknown significance, raising concerns for cancer predisposition. Given the unusual clinical presentation, somatic and germline genetic findings, and impossibility of measuring early response to therapy, the classical treatment of lymphoblastic lymphoma was modified. To minimize exposure to agents that increase DNA breakage, blinatumomab was used for consolidation. This approach led to significant tumor regression and the patient remains in remission for eight months post-diagnosis. This case underscores the importance of precise diagnosis, comprehensive somatic and germline genetic evaluation, and adapted treatment in pediatric oncology.

## 1 Introduction

This report describes a rare presentation of B-cell lymphoblastic lymphoma (B-LBL) as an isolated renal mass, initially mimicking a Wilms tumor (WT). Notably, molecular analysis of the tumor identified an atypical *EWSR1::FLI1* rearrangement, a finding previously unreported in this malignancy, along with several pathogenic variants associated with hematologic malignancies. Additionally, the patient was found to carry a germline *CHEK2* variant of uncertain significance (VUS). These findings posed a unique clinical challenge, necessitating a tailored therapeutic approach.

The atypical presentation and the inability to accurately assess early treatment response required deviation from conventional B-LBL treatment protocols. Instead of standard anthracycline- and cyclophosphamide-based intensification, which carries risks of DNA damage, the patient was treated with an antimetabolite-based regimen, with blinatumomab incorporated for consolidation. The kidney was preserved, and post-induction imaging and biopsy showed no residual tumor. The decision to use blinatumomab was influenced by emerging data from the Children's Oncology Group, demonstrating its potential to significantly improve disease-free survival in pediatric B-acute lymphoblastic leukemia (B-ALL) (1).

This case highlights the critical role of molecular diagnostics in guiding therapeutic decisions and underscores the importance of an individualized approach in managing complex and atypical pediatric malignancies.

## 2 Case description

A previously healthy 5-year-old boy presented to his primary care physician with an abdominal mass that was incidentally noticed by his mother. He was otherwise asymptomatic. Specifically, constitutional symptoms such as fever, weight loss, and night sweats were denied. He underwent abdominal ultrasound (US), which demonstrated a large left renal mass. Subsequently, a computed tomography (CT) scan revealed a large 12.7 cm×14.6 cm×12 cm left renal mass with encasement of the left renal artery. The patient was referred to the local hospital where he was noted to be hypertensive to 141/103 (>99th percentile for age) and required anti-hypertensive medication. On examination, he had an easily palpable, firm, non-tender, non-mobile mass that crossed midline and stretched from the left lower costal margin to the left lower quadrant. The family was counseled regarding a likely diagnosis of WT, and he was referred to St. Jude Children's Research Hospital for further evaluation and management.

**Figure 1:**
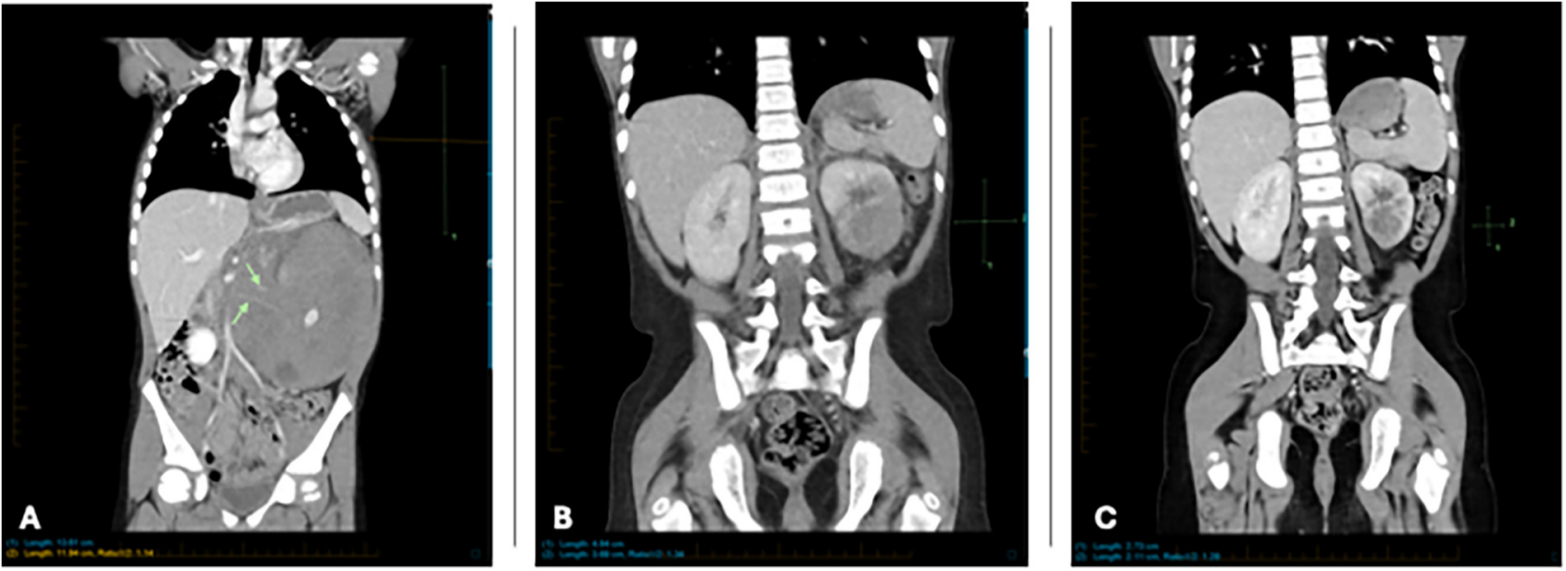
**(A)** Coronal reformatted CT at diagnosis showing large mass occupying and enlarging the entirety of the left kidney. Note encasement of renal arteries (green arrows). **(B)** Coronal reformatted CT 9 weeks after initial imaging shows significant improvement with smaller residual mass. **(C)** Coronal reformatted CT 19 weeks after initial imaging shows continued improvement with smaller residual mass.

**Figure 2:**
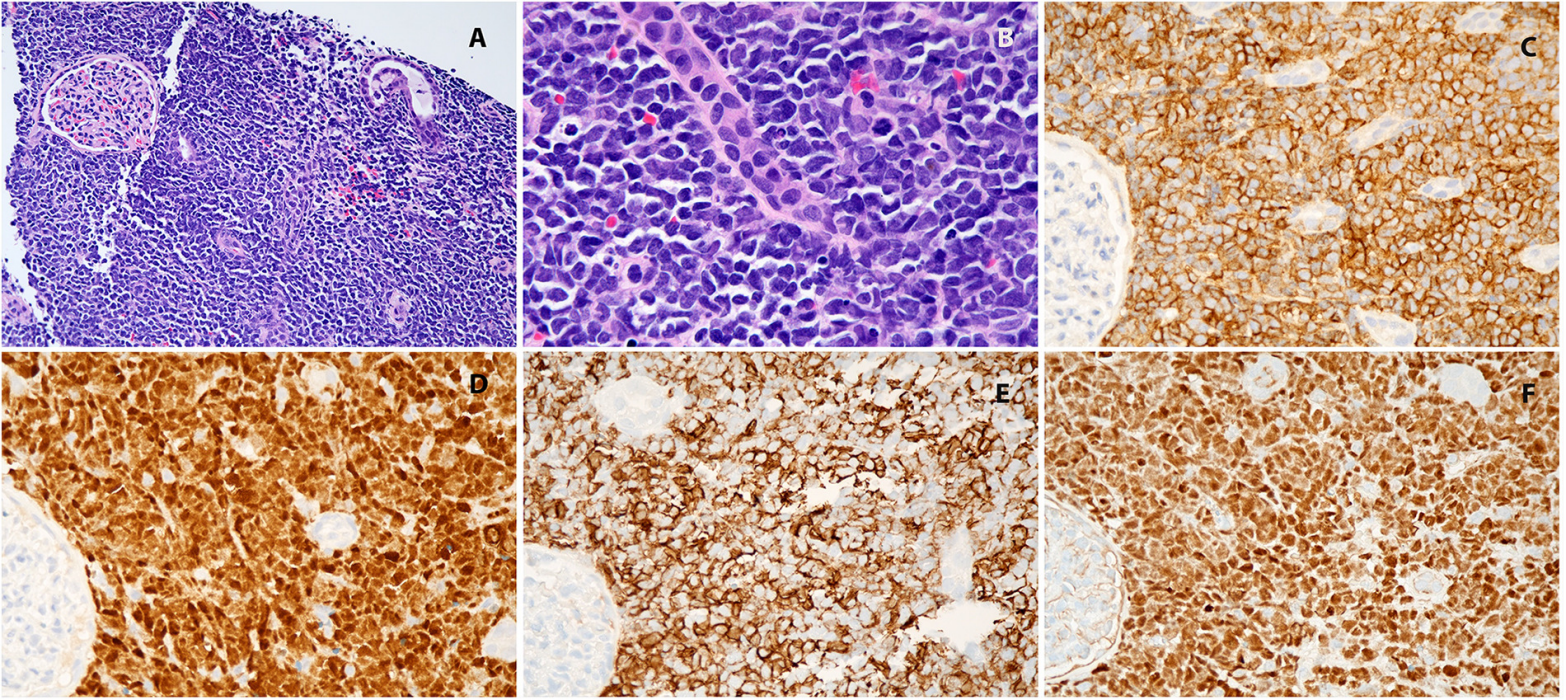
**(A)** Kidney, hematoxylin-eosin, 100×; **(B)** kidney, hematoxylin-eosin, 400×; **(C)** CD19, 400×; **(D)** TdT 400×; **(E)** CD20, 400×; **(F)** Pax5, 400×.

Upon arrival, a repeat CT of the chest, abdomen, and pelvis with contrast showed no pulmonary or nodal metastases. The scan confirmed the presence of a large left renal mass replacing most of the parenchyma of the left kidney with extrarenal extension and potential retroperitoneal lymphadenopathy. Rupture of the renal capsule could not be excluded. (Figure 1A) The patient underwent an interventional radiology (IR)-guided biopsy that revealed immature lymphoid cells/blasts replacing renal tissue. Immunostaining showed the neoplastic cells to be positive for CD45 (dim), CD19, TdT, PAX5, CD43, CD20 and negative for CD3, CD123, CD34, ALK1, EBER, NKX2.2, PHOX2B, BCOR, and WT1. In conjunction with morphology, this immunophenotype was diagnostic of B-lymphoblastic leukemia/lymphoma. (Figures 2A–F) Further diagnostic workup, including bone marrow biopsy, lumbar puncture, and a positron emission tomography (PET) scan confirmed that disease was confined to the left kidney. Cerebrospinal fluid (CSF) and bone marrow examination showed no evidence of malignancy. Results of this staging evaluation indicated the presence of Stage III disease.

The patient began standard remission induction therapy according to guidelines of the St. Jude Total 17 trial while awaiting further genetic and cytogenetic test results. Rapid RNA sequencing revealed an *EWSR1::FLI1* fusion transcript, an unusual finding for this diagnosis. Standard fusions such as *ETV6::RUNX1* and *TCF3::PBX1* were not detected. Whole exome sequencing (WES) identified somatic pathogenic or likely pathogenic variants in *WT1* and *PRDM2* with deletions within *ETV6, SETD2, ADD2, EZH2, PRDM2*, and *NF2*. Germline WES revealed a missense variant of uncertain significant (VUS) in *CHEK2*. Analysis of microsatellite instability, tumor mutational burden, and chromosomal breakage was not performed.

**Figure 3:**
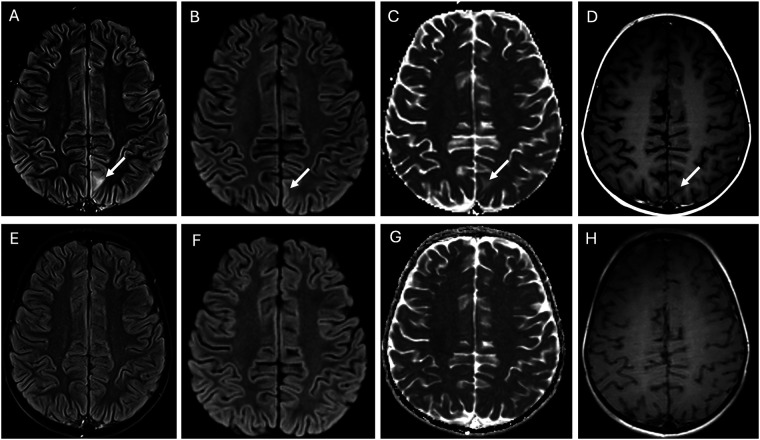
Posterior reversible encephalopathy syndrome (PRES). The top row of images at presentation shows an ill-defined area of hyperintensity in the juxtacortical region of the left paramedian parietal lobe on the FLAIR image arrow in **(A)**. This is associated with facilitated diffusion, evidenced by subtle cortical hyperintensity on the diffusion trace image arrow in **(B)** and an increased apparent diffusion coefficient (ADC) in the juxtacortical region arrow in **(C)**, consistent with vasogenic edema. Notably, there is no enhancement arrow in **(D)**. At the 4-week follow-up (bottom row images), all findings on FLAIR **(E)** and diffusion imaging **(F,G)** had completely resolved, demonstrating full reversal of the condition.

During induction therapy, the patient experienced a prolonged seizure (>1 hour), necessitating intensive care. Imaging revealed findings consistent with posterior reversible encephalopathy syndrome (PRES), likely related to his history of hypertension. Aggressive blood pressure management and anticonvulsant therapy were initiated, leading to full recovery. Subsequent imaging showed resolution of PRES-associated abnormalities. (Figure 3A–H) Treatment modifications were made to minimize anthracycline and cyclophosphamide exposure due to concerns about constitutional DNA instability. Blinatumomab was used during consolidation. A repeat PET scan after one cycle of blinatumomab showed significant regression of the left renal mass, with residual hypodense areas on CT. (Figure 1B) After a second cycle, the residual mass further decreased in size. (Figure 1C) IR-guided biopsy of the residual lesion revealed no evidence of active leukemia/lymphoma, only benign inflammatory changes and necrosis.

## 3 Discussion

Lymphoblastic lymphomas (LBL) are immature malignancies arising from T-lymphoblasts (70%–80%) and B-lymphoblasts (20%–25%) (2). According to the current World Health Organization (WHO) classification, LBL and ALL exist on a spectrum, differentiated by the degree of bone marrow involvement (3). Consequently, LBL is typically treated with protocols designed for ALL. B-LBL is predominately a disease of childhood. Most patients are diagnosed before 6 years of age, with a slight male predominance. B-LBL tends to present at a lower stage than T-LBL, and there is a decreased propensity for bone marrow involvement (4, 5). Common presentation sites include bone, manifesting as osteolytic bone lesions (26%), and skin or subcutaneous tissues (23%) (4). Less common sites include the mediastinum or pleura (11%), lymph nodes (13%), or visceral organs (4%). Among the visceral sites, the gastrointestinal tract and kidneys each account for approximately 2% of cases (6).

Wilms tumor (WT), or nephroblastoma, is the most common pediatric malignancy of the kidney, with approximately 500 new cases diagnosed annually in the United States (7). Most children present asymptomatically when a caregiver or primary care physician notices a palpable abdominal mass in a child between three and five years of age. Associated symptoms such as hypertension, hematuria, and/or flank pain occur in 20%–25% of cases (8). Mutations involving WT1 are typically seen, and several genetic syndromes such as Wilms Tumor Aniridia Genitourinary Anomalies Syndrome (WAGR), Denys-Drash, Beckwith-Wiedemann, and Frasier Syndrome have been implicated in children presenting with bilateral WT (9). Treatment approaches for Wilms tumor differ by institution. Currently, the standard of care for WT within Children's Oncology Group (COG) institutions is to perform nephrectomy at the time of diagnosis (with few exceptions) while International Society of Pediatric Oncology (SIOP) recommends nephrectomy after preoperative chemotherapy (10). Biopsy is generally avoided, as it upstages the tumor to Stage III in both staging systems.

Proceeding with treatment without histologic confirmation may result in either exposing a patient to the morbidity associated with an unnecessary surgical procedure or inappropriate administration of chemotherapeutic agents delaying appropriate therapy. While most patients treated with COG strategies will undergo primary resection, about one-fourth of patients will have IR-guided biopsy followed by chemotherapy prior to surgery. However, patients treated with the SIOP approach may not have tissue obtained for histologic confirmation of diagnosis. Biopsy is only recommended for “atypical cases”. There are defined SIOP clinical, laboratory, and radiological criteria that would lead to biopsy rather than a presumed diagnosis of WT (11, 12). (Supplementary Table 1) Although up-front nephrectomy would have been reasonable in our patient with unilateral, localized disease below the age of six, normal renal parenchyma was not visualized on diagnostic imaging leading to appropriate concern for an alternative diagnosis.

Our patient's genetic findings added further complexity. An *EWSR1::FLI1* fusion was detected, a hallmark cytogenetic abnormality in Ewing sarcoma (EWS), seen in 85% of cases. Interestingly, the fusion breakpoints in our patient differed from those typically associated with EWS (13). Two common *EWSR1::FLI1* variants are seen in EWS: Type 1 and Type 2 fusions. Type 1 fusions consist of breakpoints in exon 7 of *EWSR1* and exon 6 of *FLI1*, while Type 2 fusions consist of breakpoints in exon 7 of *EWSR1* and exon 5 of *FLI1*. These specific breakpoints bring together the transcriptional activation domain of *EWSR1* and the DNA-binding domain of *FLI1* leading to the promotion of oncogenesis. Our patient's fusion consisted of breakpoints in exon 14 of *EWSR1* and exon 4 of *FLI1*. Exon 14 of *EWSR1* encodes a domain rich in glycine and arginine, and fusions at this exon have been reported with CREM in a novel myxoid mesenchymal tumor and in a malignant epithelioid neoplasm (14, 15) The breakpoint in exon 4 of *FLI1* is part of a known breakpoint cluster region and is predicted to retain the *ETS*-type DNA-binding domain in its C-terminus (16). Although most common in EWS, *EWSR1::FLI1* fusions have rarely been described in hematopoietic malignancies including acute leukemias, myeloid sarcoma, and in secondary leukemia after a diagnosis of EWS (17–19). In addition to this atypical *EWSR1::FLI1* fusion, a heterozygous VUS in *CHEK2* was discovered in our patient. Our patient's *CHEK2* variant (*CHEK2* c.1037G > A; p.Arg346His) was a missense mutation with frequency of about 0.002% in the population in gnomAD v2.2.1 (https://gnomad.broadinstitute.org/). Although classified as a VUS, the in-silico tool REVEL predicted a deleterious effect on protein function, and functional studies have supported this predicted deleterious impact by demonstrating DNA damage induced by alkylating agents (20, 21). This variant has been documented in individuals with breast cancer (22, 23).

**Figure 4:**
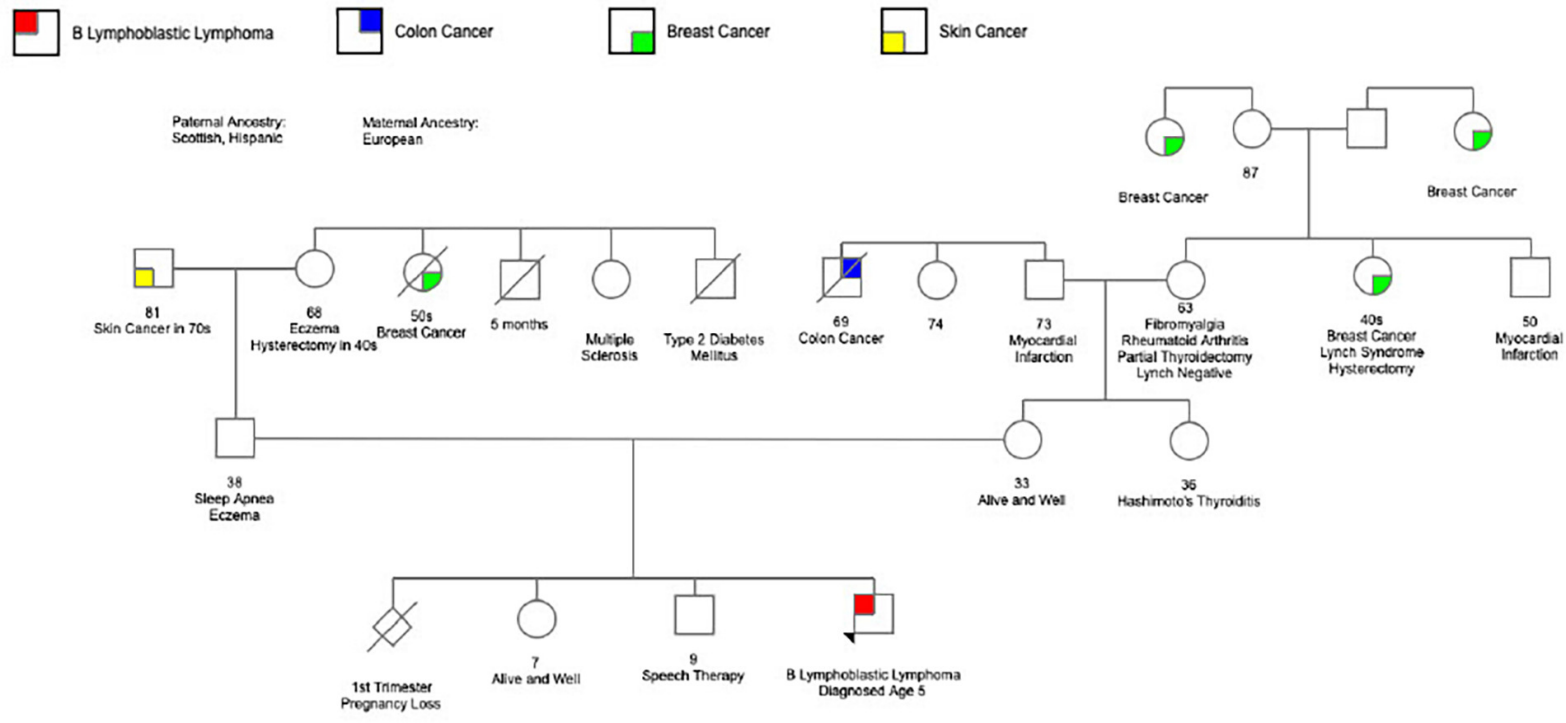
Family pedigree.

Despite the lack of definitive evidence to categorize our patient's CHEK2 variant as pathogenic, we remained concerned. Although family history was largely negative for malignancy, our patient's parents remain young. (Figure 4) The knowledge that this variant could produce deleterious effects including DNA damage resulting from exposure to alkylating agents, reports of this variant in other patients with malignancies, various coexisting somatic mutations present in the tumor sample, as well as our patient's unusual presentation left grounds for suspicion that cancer predisposition may be playing a role. Furthermore, significant limitations existed in measuring early treatment response and assessing residual disease in this case without involvement of the bone marrow. *CHEK2* encodes a serine-threonine kinase involved in DNA repair and cell cycle regulation (24). Additional anthracycline and cyclophosphamide exposure would lead to further DNA damage that our patient, with a missense variant exacting a deleterious effect on CHEK2, may not be able to adequately repair potentially raising the risk of malignancy later in life. This concern led to treatment modifications and the choice to eliminate “early intensification” (a treatment phase consisting of cyclophosphamide, cytarabine, and mercaptopurine) in favor of two cycles of blinatumomab, which would not carry the same risk of DNA damage.

In conclusion, our patient's case highlights the complex diagnostic processes and decision making often involved in the care of pediatric cancer patients. What initially appeared to be a classic presentation of WT turned out to be a rare and unexpected case of B-LBL, with an isolated renal mass as the presenting manifestation. This unusual presentation underscores the importance of considering alternative diagnoses, even when clinical and imaging findings align with more common conditions. The patient's genetic findings, including the presence of an atypical *EWSR1::FLI1* fusion and a possible cancer predisposition due to a *CHEK2* variant, further complicate the clinical picture, necessitating a more nuanced treatment approach. Ultimately, the choice to forgo traditional anthracycline-based intensification in favor of blinatumomab was influenced by the need to minimize DNA damage, given the possibility of susceptibility to future malignancies. This case emphasizes the importance of personalized medicine, genetic testing, and careful consideration of treatment strategies for patients with pediatric malignancies.

## Data Availability

The original contributions presented in the study are included in the article/Supplementary Material, further inquiries can be directed to the corresponding author.
