# Supplementary material for: Case Report: A rare pediatric case of B-cell lymphoblastic lymphoma presenting as an isolated renal mass with EWSR1::FLI1 translocation and germline CHEK2 variant
Source: Front Pediatr. 2025 Jun 24;13:1569506. doi: 10.3389/fped.2025.1569506 (PMC12234301; doi:10.3389/fped.2025.1569506)
Supplement: Supplementary file 1 [file Table1.docx]

Supplementary Table 1: SIOP Criteria for Histologic Confirmation of WT

| Clinical | Laboratory | Radiologic |
| --- | --- | --- |
| Age > 6 years | Hypercalcemia | Very large lymph nodes |
| Urinary tract infection |  | Intra-tumor calcifications |
| Septicemia |  | Nearly completely extra-renal tumor |
| Psoas inflammation/infiltration |  | No normal renal parenchyma visible |
|  |  | Lung metastasis <2 years |
|  |  | Extrahepatic and extrapulmonary metastases |
